# Environmental Ligands of the Aryl Hydrocarbon Receptor and Their Effects in Models of Adult Liver Progenitor Cells

**DOI:** 10.1155/2016/4326194

**Published:** 2016-05-04

**Authors:** Jan Vondráček, Miroslav Machala

**Affiliations:** ^1^Department of Cytokinetics, Institute of Biophysics of the Czech Academy of Sciences, 61265 Brno, Czech Republic; ^2^Department of Chemistry and Toxicology, Veterinary Research Institute, 62100 Brno, Czech Republic

## Abstract

The toxicity of environmental and dietary ligands of the aryl hydrocarbon receptor (AhR) in mature liver parenchymal cells is well appreciated, while considerably less attention has been paid to their impact on cell populations exhibiting phenotypic features of liver progenitor cells. Here, we discuss the results suggesting that the consequences of the AhR activation in the cellular models derived from bipotent liver progenitors could markedly differ from those in hepatocytes. In contact-inhibited liver progenitor cells, the AhR agonists induce a range of effects potentially linked with tumor promotion. They can stimulate cell cycle progression/proliferation and deregulate cell-to-cell communication, which is associated with downregulation of proteins forming gap junctions, adherens junctions, and desmosomes (such as connexin 43, E-cadherin, *β*-catenin, and plakoglobin), as well as with reduced cell adhesion and inhibition of intercellular communication. At the same time, toxic AhR ligands may affect the activity of the signaling pathways contributing to regulation of liver progenitor cell activation and/or differentiation, such as downregulation of Wnt/*β*-catenin and TGF-*β* signaling, or upregulation of transcriptional targets of YAP/TAZ, the effectors of Hippo signaling pathway. These data illustrate the need to better understand the potential role of liver progenitors in the AhR-mediated liver carcinogenesis and tumor promotion.

## 1. Introduction

The liver, a central organ responsible for maintaining the homeostasis in organism, plays an essential role in metabolism, both synthesizing a number of important molecules and metabolizing nutrients, xenobiotics, or various endogenous substrates [1]. It is primarily involved in glycogen storage, drug detoxification, bile production and secretion, as well as in production of serum proteins, and so forth. The metabolic and synthetic functions of the liver are performed primarily by hepatocytes, which make approximately 80% of the total liver mass [1]. Disruption of the liver capacity to detoxify, failure to secrete bile, or aberrant synthesis of plasma proteins leads to development of liver diseases, such as cirrhosis, which may ultimately result in the liver failure [2].

The liver is also an organ with a remarkable regeneration capacity that is capable of recovering both mass and function after an injury. Although hepatocytes have a very low turnover rate and under normal conditions almost all of them are quiescent cells (which reside in *G*_0_ phase of cell cycle), following liver injury, they reenter cell cycle in order to allow restoration of the original cell mass [2, 3]. Hepatocyte proliferation represents a major mechanism responsible for the liver regeneration and homeostasis [4], and, under normal conditions, the liver regeneration is thought to be primarily mediated by self-duplication of mature hepatocytes (and biliary epithelial cells) [2, 5, 6]. Nevertheless, during strong hepatocyte depletion or when hepatocyte proliferation is inhibited, the population of liver progenitor cells may serve as a second line of defense against liver injury/failure [5, 7]. The adult liver progenitor cells can give rise both to hepatocytes and to biliary epithelial cells [8, 9], although their origin, as well as their exact contribution to liver regeneration, is a matter of an ongoing debate (for recent reviews, see [2, 5–7]).

Given the importance of the liver in detoxification of xenobiotics, it is not surprising that liver cells also constitute a major target for a number of toxicants and/or their reactive intermediates. The toxic ligands of the aryl hydrocarbon receptor (AhR), such as 2,3,7,8-tetrachlorodibenzo-*p*-dioxin (TCDD) and related dioxin-like compounds (DLCs), are well-known liver toxicants, which induce multiple forms of liver damage and contribute to hepatocarcinogenesis [10]. A brief description of the AhR functions and mechanisms of the AhR-dependent signaling is also provided as a part of this review. A significant majority of currently available studies evaluating the mechanisms underlying the toxicity of the AhR agonists in the liver have so far focused on hepatocytes as a principal target of DLCs. Therefore, the primary goal of this review is to provide an overview of experimental studies evaluating the impact of toxic AhR ligands on cellular models either derived from liver progenitor cells or exhibiting phenotypic features of liver progenitors.

## 2. Adult Liver Progenitor Cells

The progenitor cells in adult liver have been considered to represent cells that may enable the liver to regenerate upon severe or chronic injury linked with impairment of proliferative capacity of hepatocytes [8]. The activation/accumulation of cells exhibiting progenitor or mixed hepatobiliary phenotypes has been observed in a number of human liver disease conditions, including submassive liver necrosis and chronic viral hepatitis, or during both alcoholic and nonalcoholic fatty liver disease [11, 12]. These cells have been proposed to represent the human equivalent of rodent oval cells (facultative liver stem cells), which are activated during a number of experimental conditions blocking the restoration of the liver mass by hepatocytes [8, 13]. The activation of oval cells in experimental animals has been documented to occur in response to a wide range of toxic insults to the liver, including (i) application of toxins/carcinogens (such as 2-acetylaminofluorene and ethionine) in combination with partial hepatectomy; (ii) use of diets containing carbon tetrachloride or 5-diethoxycarbonyl-1,4-dihydrocollidine (DDC), or (iii) using experimental choline-deficient diet supplemented with ethionine (CDE) [14–17]. These treatments lead to emergence of oval cells with bipotential ability to differentiate into both hepatocytes and biliary epithelial cells [18, 19].

The origin of rodent oval cells is still not fully clear; however, they have been hypothesized to originate from the cells that are located within canals of Hering [20]. This structure, located between hepatocytes and biliary epithelial cells, may serve as a niche for these bipotential progenitor cells; however, as will be discussed later, other origins of liver progenitor cells have also been proposed, including hepatocytes [21, 22], hepatic stellate cells [23, 24], or cholangiocytes [5]. Oval cells express markers of both the hepatocyte and bile duct lineages, including *α*-fetoprotein (AFP), delta-like 1 homolog (Dlk1), cytokeratin 19 (CK19), SRY- (sex determining region Y-) box 9 (Sox9), epithelial cell adhesion molecule (EpCAM), CD133, or MIC1-1C3 antigen [7, 8, 25]. The expression of these markers seems to be both species- and injury type-specific [25]. Therefore, additional markers of oval cells are being sought in order to improve both identification and quantification of liver progenitor cells.

The studies evaluating transdifferentiation between hepatic cell types have recently reported a number of controversial findings [2, 5–7]. Transplanted liver progenitor cells may contribute significantly to restoration of liver parenchyma, regenerating both hepatocytes and biliary epithelium [26], and the rodent liver cells expressing various markers of liver progenitors can be successfully induced to give rise to hepatocytes and/or biliary epithelial cells [27–30]. The adult bile duct derived Lgr5-positive progenitor cells have been derived and expanded from human liver, which can then be differentiated into functional hepatocytes* in vitro* or* in vivo* [31]. However, the exact contribution of adult liver progenitor cells to liver regeneration upon liver injury* in vivo* remains controversial, especially when considering the results of recent studies using genetic lineage tracing experiments. Whereas one of the first such studies has indicated that cells of biliary origin could be a major source of hepatocytes [32], others have, on the contrary, reported that adult liver progenitor cells provide only a minor fraction of cells contributing to liver regeneration, which is primarily mediated by hepatocytes under normal conditions [4, 33, 34]. Several recent studies have argued that hepatocytes arise from preexisting hepatocytes during liver regeneration or that hepatocytes within injured liver are a source of bipotential adult liver progenitors, which then contribute to restoration of hepatocyte mass through transdifferentiation [22, 35, 36]. Two recent studies have also indicated that specific progenitor/stem-like cell populations may exist in the adult liver. Recently, a preexisting population of hybrid periportal hepatocytes, expressing low levels of biliary markers, has been proposed to possess a high regenerative capacity and to contribute to restoration of liver mass after chronic hepatocyte-depleting injuries [37]. Another study has identified a population of proliferating and self-renewing Axin2-positive cells located close to the central vein within a niche established by the Wnt (wingless/integrated-1) producing endothelial cells. This population of stem cells, which is present in uninjured steady state liver, has been proposed to contribute to homeostatic liver cell renewal, similar to other organs [38].

Thus, a number of controversies currently surround both the identification of adult liver progenitor cells and their potential role(s) in homeostatic liver, during liver regeneration or in hepatocarcinogenesis. A recent study has suggested that ductular reactions may not give rise to hepatocellular carcinoma (HCC) [39], while others have proposed that dysregulated self-renewal of liver progenitor cells serves as an early event in hepatocarcinogenesis [40]. Nevertheless, regardless of the above issues concerning their origin or their role in liver regeneration, adult liver progenitor cells (which possess a significant self-renewal capacity) appear to give rise to certain types of liver cancer [41]. A significant percentage of HCC cases simultaneously exhibits both hepatocytic and biliary features [42]. A notable example is the combined hepatocholangiocarcinomas, an aggressive and heterogeneous group of liver tumors exhibiting intermediate features between hepatocytes and cholangiocytes, which have been suggested to arise from liver stem/progenitor cells [43]. This indicates that some liver cancer subtypes contain cells with phenotypic and/or functional features of liver progenitor cells, possibly originating from adult liver progenitor cell populations. Therefore, these cell populations might also constitute an important target for liver carcinogens, including the toxic environmental AhR ligands.

## 3. Toxic Ligands of the AhR and Their Hepatotoxic and Carcinogenic Effects

The AhR is a ligand-activated transcription factor, a member of the bHLH/PAS (basic helix-loop-helix/Per-Arnt-Sim) family of transcriptional regulators [44], which regulates the expression and activity of a number of genes participating in the regulation of liver cell function or hepatocarcinogenesis [10]. The AhR is in inactive state localized within the cytosolic protein complex containing chaperone protein hsp90 (heat shock protein 90), cochaperon p23, and immunophilin XAP2 (ARA9; AIP) protein. Following the binding of its cognate ligands, the AhR translocates to the nucleus and forms a dimer with the AhR nuclear translocator (Arnt). This dimer recognizes so-called xenobiotic/dioxin response elements (XRE/DRE) located within the regulatory regions of various AhR target genes. These include phase I xenobiotic metabolizing enzymes, such as cytochrome P450 family 1 (CYP1) enzymes and several phase II conjugation enzymes [45]. However, the AhR also regulates a number of genes contributing to the regulation of cell proliferation, differentiation, senescence, or programmed cell death [46–50]. This suggests that the AhR could play a major role in cell fate decisions; therefore, the aberrant long-term activation of the AhR by persistent toxic AhR ligands may contribute to important biological processes involved in hepatocarcinogenesis [51].

The AhR-null mice exhibit a number of liver defects, including reduced liver size, smaller hepatocytes, development of mild to severe liver fibrosis, accumulation of lipids, inflammation, or remodeling of the liver vascular architecture [52–54]. In some AhR knockout mice models, mild oval cells hyperplasia has been observed [52]. The ligand-dependent activation of AhR mediates toxicity of a variety of environmental pollutants, including polychlorinated dibenzo-*p*-dioxins, dibenzofurans, and biphenyls (PCBs), or polycyclic aromatic hydrocarbons (PAHs) [44]. The exposure to TCDD leads to tumor promotion, teratogenic effects, epithelial hyperplasia, thymic involution, porphyria, and (at high doses) a severe wasting syndrome followed by death of experimental animals [55]. In the rodent liver, TCDD induces a range of effects leading to hepatocellular hypertrophy, bile duct hyperplasia, formation of multinucleate hepatocytes (in some rodents), steatosis and inflammatory cell infiltration, transient liver swelling, and, at the cellular level, plasma membrane abnormalities and proliferation of endoplasmic reticulum [56]. Most of the acute toxic effects of TCDD in rodent liver are mediated by activation of the AhR in hepatocytes [54].

The AhR activation is the major and common mode of action of DLCs [10]. This allowed establishing the “toxic equivalency factor” (TEF) approach for risk assessment of mixtures containing DLCs, which is based on the concept of determination of the potencies of DLCs to activate various AhR-dependent endpoints allowing establishment of consensus TEF values (relative to TCDD) for individual DLCs [57]. Dose-additive carcinogenicity of mixtures of DLCs has been experimentally confirmed and it supports the use of the TEF approach [58].

Importantly, apart from their acute toxicity, persistent AhR ligands have been shown to act as powerful liver tumor promoters and this effect is AhR-dependent [59, 60]. TCDD and other dioxin-like compounds, such as 2,3,4,7,8-pentachlorodibenzofuran or 3,3′,4,4′,5-pentachlorobiphenyl (PCB 126), have been listed by the International Agency for Research on Cancer as carcinogenic to humans (group 1 human carcinogens) [61]. These compounds have been shown to induce multiple cancer types in experimental animals [10, 62]. The chronic toxicity and carcinogenicity of both TCDD and PCB 126 have been evaluated in two-year bioassays in female rats [63, 64]. Increased incidence of nonneoplastic liver lesions (including hepatocyte hypertrophy, altered hepatocellular foci, inflammation, oval cell and bile duct hyperplasia, cholangiofibrosis, and nodular hyperplasia) and increased incidences of hepatocellular adenomas and cholangiocarcinomas were found after exposure to DLCs. Importantly, some of these lesions also contained a prominent component of biliary epithelium and/or oval cells [65]. This suggests that liver progenitor cells might contribute to development of cancer in experimental animals exposed to DLCs.

During recent years, the AhR has been also implicated in regulation of physiological functions of stem cells of various tissue origins, in particular in hematopoietic stem cells [66–72], or in cancer stem cells [73, 74]. Importantly, a recent study has suggested that the AhR activation can have a major impact on expansion of rodent hepatic stem cells while simultaneously reducing the viability of hepatoblasts [75]. Thus, DLCs may apparently differentially affect liver cells at less differentiated stages (including adult liver progenitor cells), and their impact on liver progenitors could markedly differ from their effects in mature hepatocytes. In this review, we summarize the results of experimental studies indicating that the AhR activation may alter various functions of adult liver progenitor cells, which include deregulation of cell cycle progression/proliferation and cell-to-cell communication, as well as modulation of activities of signaling pathways regulating liver progenitor cell activation and/or differentiation.

## 4. The Role of AhR in Regulation of Cell Cycle and Proliferation in Cellular Models Derived from Hepatocytes

The mechanisms underlying the role of the AhR in carcinogenesis have recently been reviewed by several authors and it has been proposed that the AhR can play both oncogenic and tumor suppressive roles in various cancer types, in a tissue-dependent manner [76–78]. In the liver, the AhR appears to function as tumor suppressor gene in the absence of its toxic ligands [79], whereas its aberrant long-term activation induces liver carcinogenesis [10]. This is supported also by the observation that the constitutively active AhR mutant promotes carcinogenesis in mouse liver [80]. A number of mechanisms have been suggested to contribute to carcinogenic effects of DLCs in the liver, including altered proliferation of preneoplastic cells or inhibition of apoptosis leading to clonal expansion of altered hepatic foci [10, 47]. Disruption of cell proliferation control and loss of responsiveness to growth suppression belong among the hallmarks of cancer [81], which have been also suggested to contribute to carcinogenic effects of environmental chemicals [82].

The presence or absence of the AhR in cells may significantly modulate their proliferative behavior [83]. In rodent hepatoma cell models, TCDD treatment leads to AhR-dependent inhibition of *G*_0_/*G*_1_ to S-phase progression and accumulation of cells in *G*_0_/*G*_1_ phase of cell cycle [84]. This effect has been suggested to be mediated via various mechanisms including induction of the cyclin-dependent kinase inhibitor p27^Kip1^ [85], inhibition of E2F1-dependent gene expression, which is mediated by interactions between the AhR and retinoblastoma protein [86–88], displacement of E2F from the E2F-responsive promoters, or additional mechanisms dependent on AhR transcriptional partner, Arnt [89, 90]. TCDD and related compounds block cell cycle progression and cell proliferation in a majority of liver cell models used in toxicology [91, 92]. TCDD also suppresses liver regeneration following partial hepatectomy via the induction of p21^Cip1/Waf1^ activity, which is mediated by the AhR acting together with the tumor suppressor Kruppel-like factor 6, which functions as a noncanonical AhR binding partner [93, 94]. Contrary to these observations, TCDD pretreatment increases proliferative response of hepatocytes to hepatomitogen 1,4-bis[2-(3,5-dichloropyridyloxy)] benzene (TCPOBOP) in regenerating liver, which suggests that the role of the AhR in cell cycle regulation of liver cells could be more complex than simply inhibiting cell cycle progression into S-phase [95].

## 5. The Impact of AhR Agonists on Cell Cycle and Proliferation in Models of Adult Liver Progenitor Cells

Unlike in hepatocytes or hepatoma cells, various types of AhR ligands have been found to promote proliferation of rat liver progenitor cells* in vitro*. The WB-F344 cell line, isolated from the liver of adult male F344 rat, exhibits phenotypic properties of rat oval cells [96]. Upon transplantation into the liver of syngeneic rats, these cells differentiate into hepatocytes and they retain the capacity to differentiate into both biliary and hepatic lineages [97]. When WB-F344 cells are cultivated at high cell densities, under conditions of contact inhibition of cell proliferation, TCDD stimulates their proliferation [98, 99]. The contact inhibition of cell proliferation is a tightly regulated process, which restricts the cell division of confluent nontransformed cells [81]. Since tumor promotion is characterized by unbalanced proliferation either due to increased proliferation or due to decreased level of apoptosis, it has been proposed that the loss of contact inhibition is a toxic event, which may contribute to liver tumor promotion [48]. The proliferative effect of TCDD in contact-inhibited rat liver progenitor cells can be replicated also with other classes of the AhR ligands, including PCBs, polycyclic aromatic hydrocarbons, flavonoids, or endogenous AhR ligands [100–103]. The mechanism responsible for this induction of cell proliferation is strictly AhR-dependent; however, it does not depend on presence of Arnt, a transcriptional partner of the AhR [104, 105]. The proliferative effects of TCDD or other DLCs are not limited to this model of rat liver progenitor cells; similar observations have been made using some other epithelial cell models [48, 105]. In contrast, recently, the AhR activation has been found to block cell cycle progression in isolated mouse oval cells [106]. Interestingly, we have recently observed that TCDD stimulates cell proliferation in undifferentiated human liver HepaRG cells [107]. These cells, isolated from adult human liver [108], show phenotypical features of undifferentiated bipotent liver cells when cultured at low densities, and they are capable of* in vitro* differentiation towards both hepatocyte-like and biliary-like cells [109]. Together, the present data seem to indicate that, in undifferentiated liver cells exhibiting progenitor or mixed hepatobiliary phenotypes, toxic AhR ligands can induce cell proliferation, while simultaneously suppressing proliferation of hepatocytes or hepatocyte-like cells.

In the contact-inhibited rat liver progenitor cells, activation of the AhR leads to induction of JunD expression, followed by induction of cyclin A, which in turn leads to an increased activity of cyclin A/cyclin-dependent kinase 2 complex, which drives cell proliferation [105]. The disruption of contact inhibition in this liver progenitor cell model has been found to be linked also with alterations of cell-to-cell communication and modulation of signaling pathways involved both in liver regeneration and in hepatocarcinogenesis [48, 98, 107, 110–112]. TCDD has been observed to stimulate membrane translocation of c-Src kinase in WB-F344 cells [98]. This nonreceptor tyrosine kinase has been proposed to form a part of the cytoplasmic AhR complex and it significantly modulates cell behavior, including stimulation of migration and invasion of tumor cells [113–115].

Induction of cell proliferation in contact-inhibited WB-F344 cells is also associated with decreased levels of connexin 43, a major protein forming gap junctions in epithelial cells, which corresponds with reduction of gap junction plaques at cell membranes and inhibition of gap junctional intercellular communication [110]. The AhR activation in rat liver progenitor cells reduces the expression of plakoglobin (*γ*-catenin), an important constituent of desmosomes and adherens junctions; this type of regulation of plakoglobin by the AhR has been confirmed also in other cell types [112, 116]. Finally, disruption of contact inhibition in WB-F344 cells also leads to downregulation of E-cadherin (and *β*-catenin) and reduced cell adhesion, suggesting that an impaired adherens junction function could be one of the consequences of the AhR-mediated disruption of cell proliferation control [111]. All of these findings are in line with the proposed role of the AhR as a regulator of cell adhesion and cell-to-cell communication [48, 49], two key mechanisms establishing and maintaining the contact inhibition of cell proliferation [48, 117, 118]. Nevertheless, it should be stated that the* in vivo* relevance of these findings remains open and future studies should establish the importance of the AhR-dependent disruption of contact inhibition in the carcinogenic effects of its toxic ligands.

## 6. The Signaling Pathways Regulating Proliferation, Differentiation, and Fate of Adult Liver Progenitor Cells and Their Potential Interactions with the AhR Signaling

**Figure 1:**
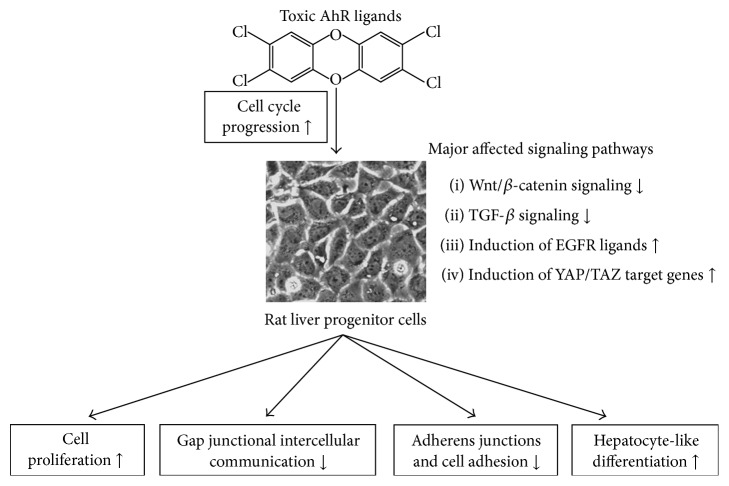
A summary of effects of AhR agonists on deregulation of signaling pathways and liver progenitor cell functions in WB-F344 cell model.

The analysis of global gene expression in WB-F344 cells released from the contact inhibition (by persistent toxic AhR ligand, PCB 126) has revealed a significant deregulation of a number of signaling components and transcriptional targets of the signaling pathways that are known to contribute to regulation of liver progenitor cell activation and/or differentiation [119]. An outline of the effects of toxic AhR ligands in this model of rat liver progenitor cell model is provided in Figure 1.

The major affected pathways included downregulation of Wnt/*β*-catenin and transforming growth factor-*β* (TGF-*β*) signaling pathways, upregulation of ligands of the epidermal growth factor receptor (EGFR), or induction of some genes regulated by transcriptional cofactor Yes-associated protein (YAP) and/or its paralogue, transcriptional coactivator with PDZ-binding motif (TAZ; also known as WWTR1), the effectors of Hippo signaling pathway.

Notably, the global gene expression data suggested that multiple members of Wnt signaling pathway can be deregulated by toxic AhR ligands in liver progenitor cells [119]. Wnt/*β*-catenin signaling pathway is a key pathway regulating both development and adult tissue homeostasis. In the absence of Wnt stimulation, *β*-catenin binds to cytoplasmic destruction complex, which is formed by tumor suppressor proteins Axin and adenomatous polyposis coli, and kinases belonging to glycogen synthase kinase 3 and casein kinase 1 families. Within this complex, *β*-catenin is phosphorylated, ubiquitinated, and consequently degraded via a proteasome [120–122]. The activation of cognate Wnt receptor (Frizzled) and coreceptor (low density lipoprotein 5/6) by Wnts or related ligands inactivates the cytoplasmic destruction complex, thus leading to accumulation and nuclear translocation of *β*-catenin. The nuclear *β*-catenin forms complexes with LEF (lymphoid enhancer-binding factor)/TCF (T-cell factor) transcription factors and drives transcription of their target genes in a cell context-specific manner [120–122]. Apart from its signaling role, *β*-catenin plays also an important structural role in formation of adherens junctions, main epithelial adhesive junctions further contributing to the regulation of *β*-catenin activity and turnover [123]. In the liver, Wnt/*β*-catenin signaling plays a major role in its embryonic development, early postnatal growth, regeneration, and maintenance of adult liver functions, such as liver zonation (for recent reviews, see [124, 125]). Deregulation of Wnt signaling is also an important factor in liver carcinogenesis [125]. Importantly, a number of reports have indicated that the activity of this pathway controls proliferation and/or differentiation of liver progenitor cells, as well as liver cancer stem cells [40, 126–131].

It is becoming increasingly evident that Wnt/*β*-catenin signaling interacts with the AhR at multiple levels [132]. The increased activity of *β*-catenin upregulates the AhR expression in various tissues, including mouse liver [133, 134]. Given that *β*-catenin activity is high within the pericentral zone [135, 136], this may imply that the cells surrounding the central vein (including the proposed liver stem cells contributing to hepatocyte renewal [38]) could be more sensitive to toxic AhR ligands, because of significantly higher AhR levels in this region, as compared with periportal zone [137]. *β*-Catenin plays a major role in the expression of xenobiotic metabolizing enzymes in the liver [133, 136]. These include in particular the CYP1 family enzymes regulated by the AhR, which are involved in bioactivation of numerous environmental carcinogens [45, 138–141]. In rat liver progenitor cells, activation of Wnt/*β*-catenin has been found to significantly promote expression of both Cyp1a1 and Cyp1b1 [111] playing a principal role in bioactivation and genotoxicity of the environmental AhR ligands, such as PAHs [45].

Importantly, at the same time, TCDD could block the *β*-catenin-dependent signaling in liver progenitor cells. WB-F344 cells are sensitive to the activation of the canonical Wnt signaling by recombinant Wnt ligands, which induce a moderate cell proliferation in this cell model [101, 142]. The activation of AhR by TCDD significantly decreases levels of the active form of *β*-catenin (dephosphorylated on S37 and T41 residues) in liver progenitor cells [111]. Downregulation of *β*-catenin by activated AhR has also been observed in other cell models [143]. Additionally, TCDD induces dephosphorylation of Dvl (dishevelled) 2 and Dvl3 proteins that play a key role as the branching points regulating both canonical and noncanonical Wnt pathways [144]. In line with this, the sustained AhR activation has been found to reduce expression of a number of Wnt/*β*-catenin pathway targets in WB-F344 cells [111, 119].

The AhR-mediated deregulation of Wnt/*β*-catenin-dependent transcription in WB-F344 has been also linked with changes in their progenitor phenotype since CK14 and CK19, which are abundantly expressed in oval cells [145, 146], are downregulated by TCDD, while CK8 is simultaneously upregulated, which suggests that progenitor cells exposed to the AhR ligands may progress towards more hepatocyte-like phenotype [111]. This is supported also by the observation that expression of other genes associated with hepatic progenitor cell compartment, such as Kitl and Ncam1, is downregulated in WB-F344 cells exposed to toxic AhR ligands [111, 119]. The inhibitory role of the AhR in this important signaling pathway, contributing to regulation of progenitor cell proliferation and phenotype, is also supported by additional studies indicating that AhR ligands repress production of canonical Wnt ligands and/or repress Wnt/*β*-catenin signaling in a variety of tissues and cell models, including embryonic stem cells [143, 147–151]. Together, these results indicate that, apart from modulating the structural role of *β*-catenin in adherens junctions, toxic AhR ligands might also block its signaling role in liver progenitors.

Wnt/*β*-catenin signaling is closely connected with the TGF-*β* signaling pathway, since both pathways cross-talk at multiple levels, such as through reciprocal regulation of their ligands or via direct interaction of their signaling effectors within cell nuclei that are involved in transcriptional regulation of common gene targets [152, 153]. The TGF-*β* family of cytokines includes, apart from TGF-*β*1, a number of proteins playing important roles in embryonic development, adult tissue homeostasis, and the cancer development, such as bone morphogenic proteins and activins/inhibins. TGF-*β*1 is a pleiotropic cytokine inducing a range of effects within liver cells, including regulation of cell migration, invasion, and stemness [154]. TGF-*β*1 blocks proliferation and induces apoptosis in mature hepatocytes, while its role in liver progenitor cells is less clear [154, 155]. Some studies have indicated that adult liver progenitor cells could be less sensitive to the TGF-*β*1-induced apoptosis and its antiproliferative effects than hepatocytes [156–158]. On the other hand, TGF-*β*1 blocks proliferation and promotes apoptosis in oval cell lines derived from DDC-treated mice [17]. Active TGF-*β*1 and *β*3 proteins are elevated in AhR knockout mice, and this corresponds with increased numbers of hepatocytes undergoing apoptosis, as compared with wild-type mice [159]. Interestingly, the analysis of global gene expression changes in WB-F344 cells revealed that AhR ligands could induce expression of follistatin in rat liver progenitors [119]. Upregulation of follistatin by TCDD has been observed also in additional cell models [160, 161]. This protein directly binds and inhibits activin A, TGF-*β* family member and regulator of the liver homeostasis. Activin A blocks hepatocyte proliferation and induces their apoptosis, while its inhibition via follistatin promotes proliferation and decreases apoptosis in the liver [162]. Since activin A has been shown to induce growth arrest in hepatic progenitor cells [163], its inhibition via the AhR-dependent induction of follistatin might further promote the proliferative effects of toxic AhR ligands in rat liver progenitors. Additionally, AhR ligands have been also observed to downregulate activin receptors, which may pronounce their impact on activin signaling [119].

Growth factor signaling plays an important role in regulation of oval cells response, as it contributes to regulation of their growth, survival, motility, and differentiation [154, 155]. Rat liver progenitor WB-F344 cells are sensitive both to hepatocyte growth factor (HGF) and to epidermal growth factor (EGF), which both stimulate their proliferation and/or protect them from apoptosis [100, 164]. EGFR can be activated, apart from EGF, also by other functionally related ligands, such as TGF-*α*, heparin-binding EGF (HB-EGF), amphiregulin (Areg), and epiregulin. The EGFR ligands are upregulated during oval cell activation and they promote oval cell expansion* in vivo* [165, 166]. Oval cell lines have been also proposed to regulate EGFR signaling also via autocrine mechanism(s) [154]. The treatment of WB-F344 cells with toxic AhR ligands has been found to upregulate several EGFR ligands, including Areg and HB-EGF, in the AhR-dependent manner [119]. Areg is a candidate AhR-responsive gene, which has been found to be induced by AhR ligands (or their mixtures) in the developing ureter* in vivo*, as well as in mouse hepatoma and human oral epithelial cells* in vitro* [167, 168]. Whether expression of Areg is increased in response to TCDD in adult liver progenitor cells also* in vivo* remains to be determined. HB-EGF is another EGFR ligand, which has been shown to contribute to liver regeneration or hepatocarcinogenesis [169–171]. TCDD has been shown to regulate also expression of additional EGFR ligands, such as epiregulin or transforming growth factor-*α* [172, 173]; however, these have not been found to be upregulated in WB-F344 cells [119]. Taken together, rat liver progenitor cells are capable of the AhR agonist-inducible production of some EGFR ligands; however, the functional role of induction of these growth factors upon AhR activation is not fully clear.

The Hippo signaling pathway is essential for a proper organ size control, tissue regeneration, and stem cell self-renewal and it can play a significant role in cancer development [174]. In mammals, this pathway consists of a core set of kinases, mammalian Ste2-like kinases 1/2 (Mst1/2), and large tumor suppressor kinases 1/2 (Lats1/2), which control the activity of YAP and TAZ [175]. The establishment of cell-cell contacts leads to activation of Hippo kinases, Mst1/2 and Lats1/2, which then inhibit YAP and/or TAZ activity via their cytoplasmic retention and/or proteasomal degradation [175]. In contrast, activation of cell proliferation is linked with active YAP and/or TAZ being present within the nucleus, where they control the expression of a number of growth-promoting or antiapoptotic genes, including connective tissue growth factor (CTGF), cysteine-rich angiogenic inducer 61 (CYR61), or survivin [176, 177]. The activity of proteins constituting Hippo pathways overlaps at numerous points with other pathways controlling the activation of adult liver progenitor cells, such as Wnt/*β*-catenin signaling [178], and it is a principal regulator of contact inhibition [177, 179]. In the liver, knockout of Hippo pathway components regulating YAP/TAZ activity or overexpression of YAP leads to disruption of liver size control or development of HCC [180]. The Hippo pathway has been also proposed to contribute to bile duct development and to hepatocyte reprogramming to biliary epithelial cells [181, 182]. Toxic compounds such as TCBOPOP have been shown to simultaneously increase liver size and increase YAP levels in liver [183]. Both YAP and TAZ have been shown to modulate plasticity and differentiation of hepatocytes, to control development of cancer stem cells during HCC, or to modulate proliferation of HCC cells, thus indicating a potentially important role for Hippo pathway in hepatocarcinogenesis [181, 184–186].

At present, the information on interactions between the AhR activation and Hippo signaling is very limited. Interestingly, the disruption of contact inhibition in rat liver WB-F344 cells has been found to be associated with induction of some YAP/TAZ transcriptional targets [107, 119]. Survivin, which is regulated by YAP/TAZ [187], has been found to be upregulated by TCDD in undifferentiated human liver HepaRG cells, simultaneously with disruption of cell cycle control and induction of cell proliferation [107]. These results seem to indicate that activation of proliferative signaling in cellular models of liver progenitors could be linked with YAP/TAZ-dependent activation of some target genes of this pathway; however, at present, the* in vivo* relevance of these findings remains unclear. Nevertheless, it is of interest that both TCDD and PCB 126 induce expression of CTGF, another YAP/TAZ transcriptional target, in contact-inhibited rat liver progenitor cells [107, 119]. This protein has been shown to promote hepatocyte-like differentiation of rat liver progenitor cells* in vitro* [188]; this seems to support the observation that AhR ligands reduce expression of progenitor cell markers and increase levels of hepatocyte-like markers in rat liver progenitors [111]. Nevertheless, TCDD has been also found to repress CTGF mRNA in HL1-1 adult human liver stem-like cell line, while simultaneously inducing YAP mRNA in the same cell model [189], thus suggesting that the role of the AhR in regulation of CTGF could be more complex and perhaps cell-specific.

Inflammatory cytokines have been proposed to play a major role in mediating both the hepatotoxicity of TCDD and the TCDD-induced liver tumor promotion [59, 190]. The production of inflammatory cytokines, such as tumor necrosis factor-*α* (TNF-*α*), also contributes to the expansion of oval cells during experimental rodent liver injury [191], since TNF-*α* is upregulated during oval cell proliferation, which is induced by CDE diet, and elimination of TNF receptor 1 blocks oval cell response in mice [192]. Interestingly, TNF-*α* has been found to be a major factor supporting the proliferative effects of DLCs in rat liver oval cells, promoting both cyclin A induction and cell cycle progression in WB-F344 cells [193]. This cytokine potentiates the effects of both strong and weak environmental AhR agonists in rat liver progenitor cells [193, 194]. Moreover, activation of inflammatory signaling by this cytokine, namely, the p38 mitogen-activated kinase activity, further supports inhibition of gap junctional intercellular communication [194], as well as metabolic activation of genotoxic AhR ligands via upregulation of CYP1B1 [195, 196]. Together, these data seem to support the scenario, where induction of inflammation by toxic AhR ligands in the liver might further support their procarcinogenic effects (deregulation of cell proliferation, induction of DNA damage) in liver progenitors.

## 7. Conclusions

Toxic AhR ligands have been found to disrupt various functions of liver progenitor cell models* in vitro*, including deregulation of cell proliferation control and cell-to-cell communication, or to alter the activity of signaling pathways relevant for the maintenance of liver homeostasis, activation of oval cell response, or liver carcinogenesis. These include modulations of Wnt/*β*-catenin and TGF-*β* pathways and induction of expression of EGF-related growth factors or transcriptional targets of Hippo pathway, which are involved in both regenerative and oncogenic signaling. The available data seem to indicate that, apart from their other well-recognized hepatotoxic effects, the environmental ligands of the AhR may alter the functions of cell populations exhibiting phenotypic features of adult liver progenitor cells (or undifferentiated liver cells), with potential to serve as precursors of hepatocytes and biliary epithelial cells. These results may have implications for the carcinogenic effects of sustained AhR activation in the liver; however, it should be noted that a major limitation of the available data is currently their reliance on the use of* in vitro* models of liver progenitor cells. Future studies should therefore focus on analyzing the impact of the AhR activation on liver progenitors* in vivo*, in order to ascertain the relevance of these findings for experimental chemical carcinogenesis, which may help us to better understand the liver toxicity and carcinogenicity of the AhR ligands, both in the experimental animals and in humans.
